# The impact of male sex on outcomes in primary biliary cholangitis

**DOI:** 10.1038/s41598-026-44615-0

**Published:** 2026-03-31

**Authors:** Nirbaanjot Walia, Julian Pohl, Matthias Reinhardt, Charalampos Pavlidis, Janine French, Münevver Demir, Frank Tacke, Cornelius Engelmann

**Affiliations:** 1 Department of Hepatology and Gastroenterology, Charité—Universitätsmedizin Berlin, Campus Virchow-Klinikum (CVK) and Campus Charité Mitte (CCM), Augustenburger Platz 1, 13353 Berlin, Germany; 2 Department of Gastroenterology and Hepatology, Austin Health, 145 Studley Rd, Heidelberg, VIC 3084 Australia; 3 Biostatistics Unit, Melbourne School of Population and Global Health, The University of Melbourne, Melbourne, 207 Bouverie St, Carlton, VIC 3053 Australia; 4 Institute for Liver and Digestive Health, Royal Free Campus, University College London, Rowland Hill Street, London, NW3 2PF UK

**Keywords:** Liver transplant, Mortality, Death, Decompensation, Autoimmune, Cholestatic disease, Cholestasis, Gender difference, Complications, Liver-related complications, Diseases, Gastroenterology, Medical research, Risk factors

## Abstract

Limited evidence suggests men with Primary Biliary Cholangitis (PBC) experience worse outcomes than women. The aims of this study were to assess sex-related differences in outcomes in PBC, and to determine whether differences were more specific to PBC than other cirrhosis aetiologies. This study conducted at Charité Berlin analysed inpatient admissions from 2011 to 2022 with PBC. The primary outcome was a composite of inpatient mortality or liver transplant. Secondary outcomes included inpatient mortality, liver transplant, decompensated cirrhosis, and hospitalisation duration. Univariable and multivariable logistic and linear regression assessed the impact of sex on outcomes. This was compared to a control group of cirrhosis patients, matched on age and co-morbidities on a 1:1 ratio. 18% of the 940 inpatient admissions with PBC were male. Men had higher rates of liver-related complications, were younger than women, and had more deranged liver function tests. Men had an increased odds of the primary outcome on univariable analysis (OR = 4.28, 95% CI: [2.52–7.23], *p* < 0.001) and following adjustment for age and comorbidities (OR = 3.68, 95% CI: [2.06–6.58], *p* < 0.001). This contrasted with the control group, where men had a reduced odds of death or transplant on univariable (OR = 0.65, 95% CI: [0.44–0.98], *p* = 0.036) and multivariable (OR = 0.63, 95% CI: [0.42–0.96], *p* = 0.030) regression. Interaction analysis across the combined cohort revealed a modification effect of PBC on the risk of the primary outcome by sex (OR = 6.59, 95% CI: [3.39–12.75], *p* < 0.001). Similar findings were noted for secondary outcomes. Men with PBC may experience worse outcomes than women. Similar differences are not apparent for other cirrhosis aetiologies.

**Supplementary Information:**

The online version contains supplementary material available at 10.1038/s41598-026-44615-0.

## Introduction

Primary Biliary Cholangitis (PBC) is a chronic autoimmune liver disease characterized by progressive destruction of intrahepatic bile ducts^1,2^. If left untreated, patients may experience worsening cholestasis, cirrhosis and eventual liver failure. PBC primarily affects females, with traditional studies reporting female-to-male ratios varying from 9:1 to as high as 22:1^3–6^. Recent analyses have demonstrated a rising prevalence of PBC, with increasing proportions of PBC patients being male^7–9^, perhaps owing to a greater recognition of this disease, particularly in males.

Despite the lower prevalence of PBC in men, there is evidence to suggest male patients may experience more severe disease progression and poorer outcomes compared to females, especially with regards to mortality^7,8,10,11^. However, mortality as an outcome, is limited in its ability to demonstrate more severe progression of PBC in males.

There is a relative paucity of data assessing sex-related differences in outcomes which would suggest a more severe progression of PBC in males, such as decompensation events or other complications associated with liver disease^12,13^. It is also unclear whether any sex-related disparities are specific to PBC, or a feature of other forms of chronic liver disease, as these studies often do not compare sex-based differences in outcomes in PBC to non-PBC cohorts.

Moreover, the predominance of females in PBC studies has led to relatively small male sample sizes in many studies, limiting the generalisability of findings. As such, this study aims to further assess the impact of male sex on outcomes in PBC, and to determine whether any differences in sex related outcomes are specific to PBC compared with other causes of chronic liver disease.

## Methods

### Study design, population and data collection

This retrospective, observational study included inpatient admissions at Charité - Universitätsmedizin in Berlin, Germany from 2011 to 2022. International Statistical Classification of Diseases and Related Health Problems – German Modification (ICD-10-GM) codes were used to identify and include inpatient admissions with PBC, as well as other aetiologies of cirrhosis for comparison, irrespective of the primary reason for admission. Similarly, ICD-10-GM codes were used to identify comorbidities and various complications of liver disease (see Table S1). Operation and Procedure (OPS) code ‘5–504’ was used to identify patients who underwent liver transplantation. Additional data available included age, sex, duration of admission, inpatient blood test results and inpatient mortality. Sex was recorded based off patient self-reporting. This retrospective study protocol was reviewed and approved by the institutional ethics committee of the Charité Berlin (ethical approval number EA1/276/24) and was carried out in accordance with the Declaration of Helsinki and Istanbul.

### Outcomes

The primary outcome of interest was a composite of inpatient mortality and liver transplantation during the recorded admission episode. Secondary outcomes included duration of admission, complications of liver disease, hepatic decompensation, liver transplantation and mortality during the admission. Episodes of decompensation were defined by the presence of hepatic encephalopathy (HE), variceal haemorrhage (VH), hepatorenal syndrome (HRS), ascites or spontaneous bacterial peritonitis (SBP). Other complications of liver disease were defined by portal venous thrombosis (PVT), hepatocellular carcinoma (HCC), sarcopenia, osteoporosis and malnutrition.

### Statistical analysis

Statistical analyses were conducted using R software version 4.3.2. Differences between groups were assessed using Mann-Whitney U tests for continuous variables, and Fisher’s Exact or Chi-Square tests for categorical variables. Univariable and multivariable logistic regression was used to assess the impact of male sex on the above binary outcomes in PBC. Linear regression was used to assess duration of admission – which was log-transformed to achieve a normal distribution.

To determine whether sex-related differences were specific to PBC, these data were compared to a control group of patients with alternate cirrhosis aetiologies, matched by age and comorbidities with a 1:1 ratio using nearest-neighbour propensity score matching, with an interaction term for sex and PBC included in the modelling. Matching was performed on a cohort of 13,023 admissions with an ICD code for cirrhosis, with aetiologies such as alcohol-related liver disease (ALD), metabolic dysfunction associated-steatotic liver disease (MASLD), hepatitis C (HCV) and hepatitis B (HCV). Adequacy of matching was determined by standardised mean differences, variance ratios and empirical cumulative distribution functions statistics.

Several post-hoc sensitivity analyses were conducted. Generalised estimating equations (GEEs) were used to account for any correlations between potential repeated admissions for the same patient to assess the consistency of any sex-related differences on outcomes observed in PBC. Regression analysis was repeated in a higher-specificity PBC subset given PBC identification on an ICD-10 code alone may result in misclassification. Inclusion into this subset was defined by ≥ 2 PBC ICD-10-GM codes and/or elevated alkaline phosphatase and/or supportive serology where available (anti-mitochondrial antibody, SP100 and/or GP210), with exclusion of concurrent autoimmune hepatitis cases. Finally, analyses were repeated in a propensity score–matched cohort in which all male admissions were matched 1:1 to female admissions using nearest-neighbour matching based on age and comorbidities.

## Results

### Baseline characteristics

A total of 940 PBC inpatient admissions were identified, with 171 (18.2%) being male (Table 1). The median age of men was 57.5 years (IQR 37.7–69.7), which was younger than women, who had a median age of 61.5 years (53.4–70.9), *p* < 0.001). Men had lower rates of obesity (2.3% vs. 6.8%, *p* = 0.03), but higher rates of chronic kidney disease (CKD, 14.6% vs. 8.7%, *p* = 0.023). Men also had lower rates of ischaemic heart disease, congestive heart failure and chronic obstructive pulmonary disease (COPD), and higher rates of diabetes, but these differences were not statistically significant. Among male admissions, 7 of 171 (4.1%) had a diagnosis of autoimmune hepatitis compared with 72 of 769 female admissions (9.4%).

The matched non-PBC cohort consisted of 940 patients, with aetiologies including ALD (*n* = 787), HCV (*n* = 90), HBV (*n* = 44) and MASLD (*n* = 60). As can be seen in Tables S2 and S3, matching was balanced across age and comorbidities. There were no significant differences in age and comorbidities between males and females in this cohort.

**Fig. 1:**
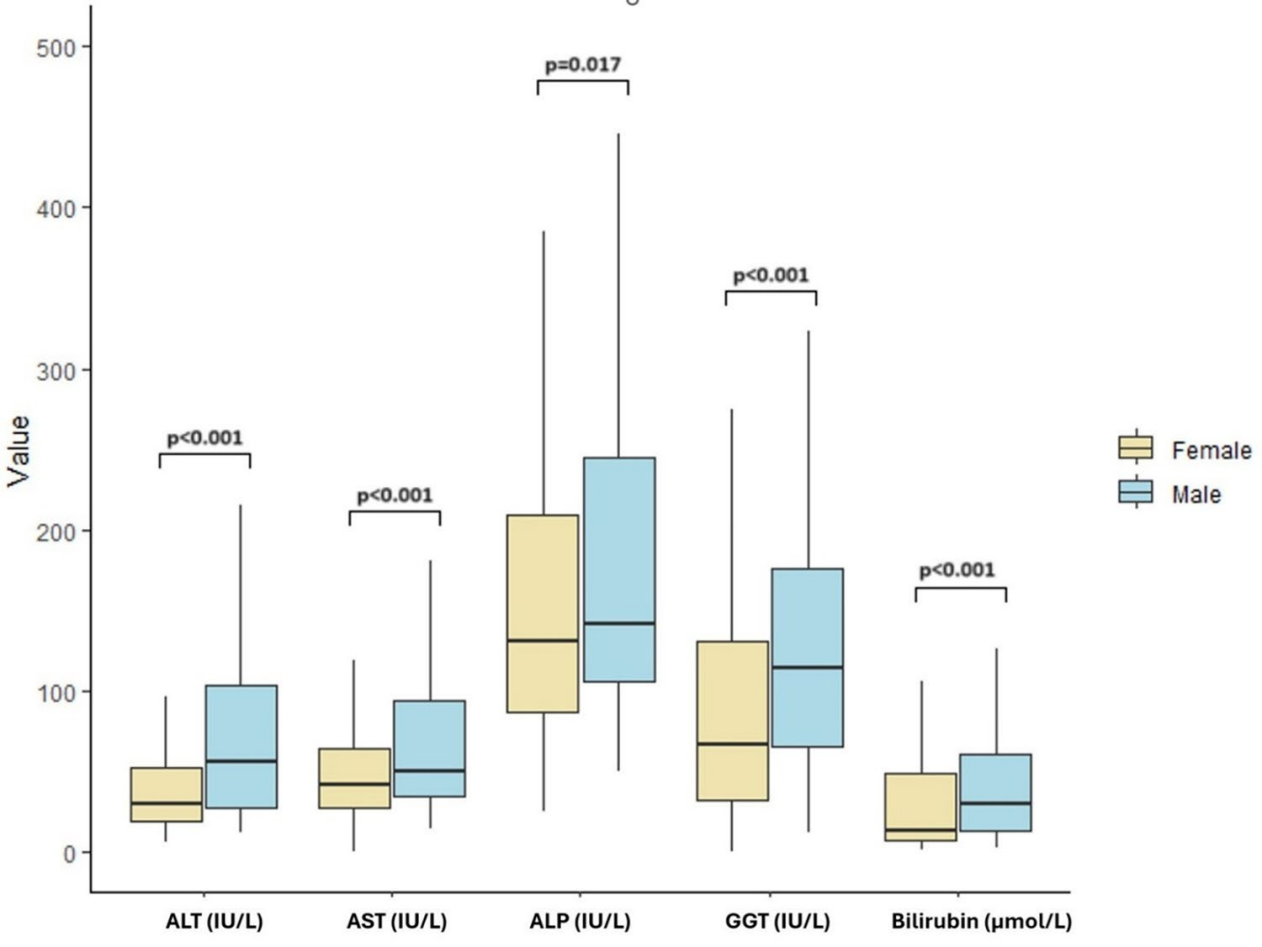
Sex-based comparison of baseline liver biochemical parameters in the PBC cohort. Boxplots show baseline levels of alanine aminotransferase (ALT), aspartate aminotransferase (AST), alkaline phosphatase (ALP), gamma-glutamyltransferase (GGT), and total bilirubin stratified by sex. Boxes indicate the interquartile range (IQR) with the median shown as a horizontal line; whiskers extend to 1.5×IQR. Male patients demonstrated significantly higher levels of ALT, AST, GGT, and bilirubin compared with females (all *p* < 0.001), while ALP levels were also higher in males (*p* = 0.017). *p*-values indicate comparison between sexes.

With regards to pathology and liver function tests results on admission, men with PBC had significantly higher median ALT, AST, ALP, GGT, INR and bilirubin compared to women (Fig. 1). Men also had lower platelet counts compared to women. No significant differences were observed in the non-PBC cohort except for a higher ALT.

In PBC, MELD (Model for End-Stage Liver Disease) and MELD-Na scores were also significantly higher in males compared to females (Table 1), whilst the non-PBC cohort had similar MELD and MELD-Na scores between males and females. Median MELD 3.0 scores were also higher in PBC males compared to females, however this difference was not statistically significant. In contrast, in the non-PBC cohort, MELD 3.0 was significantly lower in males than females. While there were similar proportions of Child-Pugh A assignment (18.7% for men and 18.1% for women) in PBC, there were significantly higher proportions of men with Child-Pugh B (13.5% vs. 6.9%, *p* = 0.007) and Child-Pugh C (8.2% vs. 1.4%, *p* < 0.001) cirrhosis. In the non-PBC cohort, Child-Pugh class assignments did not differ between the sexes.

### Complications of liver disease

Men with PBC demonstrated a consistent numerical trend towards higher rates of all complications of liver disease assessed, apart from osteoporosis. These differences were significant for bacterial peritonitis (6.4% vs. 2.7%, *p* = 0.032), portal vein thrombosis (PVT, 3.5% vs. 1.0%, *p* = 0.028) and malnutrition (6.4% vs. 2.7%, *p* = 0.032), while remaining differences were smaller and not statistically significant. By contrast, in the non-PBC cohort, men had lower rates of all complications apart from hepatocellular carcinoma (HCC, 22.4% vs. 9.0%, *p* < 0.001).

**Fig. 2:**
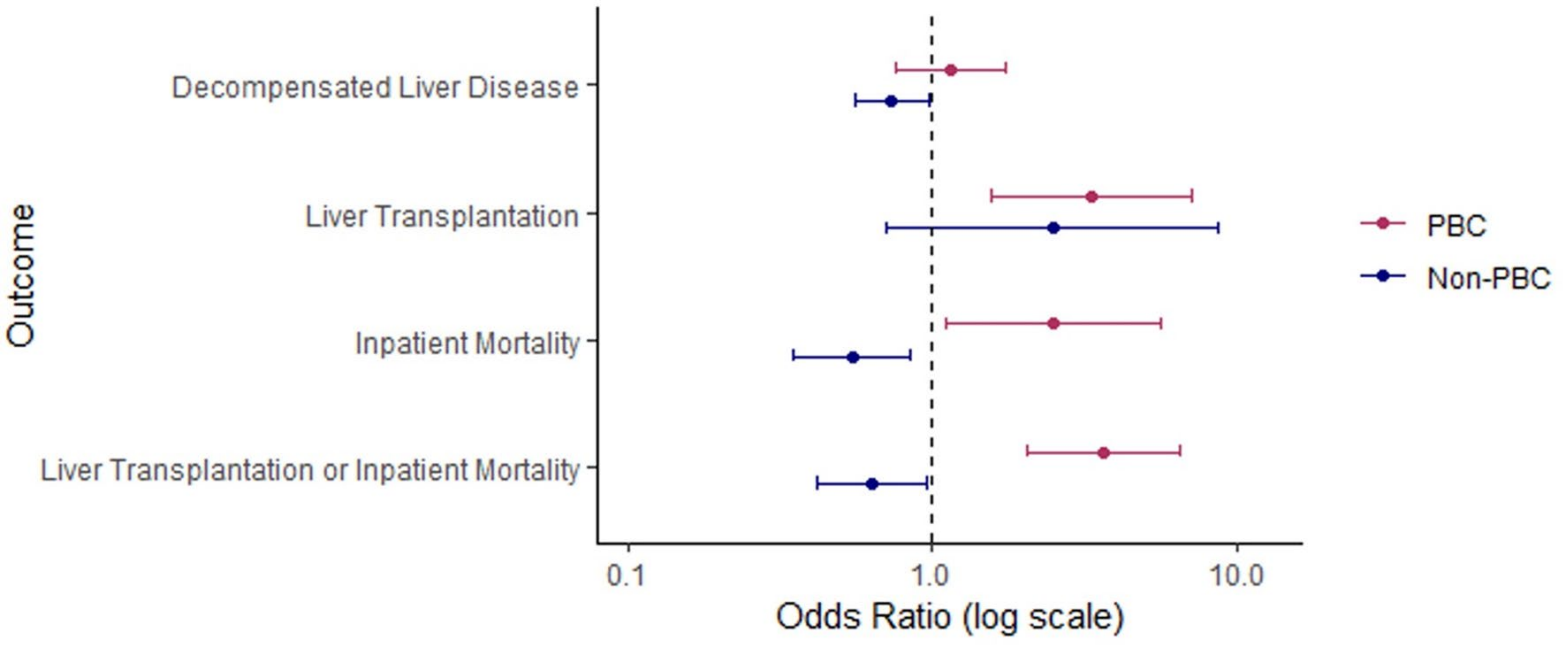
Adjusted odds ratios for adverse inpatient outcomes associated with male sex in the PBC and non-PBC cohorts. Forest plot showing adjusted odds ratios and 95% confidence intervals for decompensated liver disease, liver transplantation, inpatient mortality, and the composite outcome of liver transplantation or inpatient mortality. Models were adjusted for age and comorbidities. The dashed vertical line indicates an odds ratio of 1.0.

Univariable analysis demonstrated that men had increased odds of meeting the primary composite outcome of inpatient death or transplantation in the PBC cohort (OR = 4.28, 95% CI: [2.52–7.23], *p* < 0.001) (Table 2). These findings persisted when adjusted for age and comorbidities (adj. OR = 3.69, 95% CI: [2.06–6.58], *p* < 0.001) (Table 3; Fig. 2). The opposite was seen in the non-PBC cohort, with men being at decreased odds of meeting the primary outcome before and after adjustment (adj. OR = 0.63, 95% CI: [0.42–0.96], *p* = 0.030).

Similar findings were noted for secondary outcomes on univariable and multivariable analysis. Following adjustment, men had longer lengths of admissions (adj. β = 0.17, 95% CI: [0.02–0.33], *p* = 0.028) and increased odds of inpatient mortality (adj. OR = 3.38, 95% CI: [1.08–5.59], *p* = 0.027) and of requiring liver transplantation (adj. OR = 3.38, 95% CI: [1.57–7.26], *p* = 0.002). They also had increased odds of decompensation (adj. OR = 1.145, 95% CI: [0.75–1.73], *p* = 0.53), however this was not significant.

In the non-PBC cohort, men had reduced odds of inpatient mortality (adj. OR = 0.54, 95% CI: [0.35–0.85], *p* = 0.006) and decompensation (adj. OR = 0.74, 95% CI: [0.55–0.98], *p* = 0.0340), but had non-significantly higher odds of liver transplantation (adj. OR = 2.47, 95% CI: [0.78–10.17], *p* = 0.158). Men also demonstrated shorter lengths of admission (adj. β=−0.10, 95% CI: [−0.24–0.04], *p* = 0.181), but this was not significant.

The interaction analysis across the combined cohort revealed a modification effect of PBC on the risk of the primary outcome by sex (OR = 6.59, 95% CI: [3.39–12.75], *p* < 0.001), as well as all secondary outcomes apart from liver transplantation (Table 4).

Additional sensitivity analyses were conducted to further evaluate these results and are available in the Supplementary Materials. First, GEE modelling to account for within-subject clustering demonstrated consistent findings (Table S4). Second, restriction to a higher-specificity PBC cohort (defined by multiple diagnostic codes for PBC, and/or elevated ALP, and/or serological evidence where available) yielded similar trends (Table S5). This PBC sub-cohort included a total of 121 male and 573 female cases, and additionally excluded any remaining AIH cases. Third, analyses on a PBC cohort where males were matched to females on a 1:1 ratio on age and comorbidities was conducted. This yielded similar results for the primary outcome and consistent trends for the secondary outcomes. Balance of matching and regression results are presented in Tables S6 and S7.

## Discussion

PBC remains the most common acquired cholangiopathy^1^, with a rising prevalence and increasing recognition of the disease in men^7,8^. This study aimed to determine whether male sex led to worse outcomes in PBC patients, and whether any sex related differences were specific to PBC rather than chronic liver disease in general. Males admitted to hospital with PBC were consistently found to have more severe disease as measured by the degree of serum blood test derangement and rates of complications.

Men had significantly greater liver function derangement, including higher ALT, AST, ALP, GGT and bilirubin levels on admission compared to women, which is consistent with previous findings by Muratori et al.^14^. Despite men having higher upper limits of the normal range of liver function test results^15^, differences to this degree weren’t expected, especially when compared against the relatively smaller differences in male and female blood test results in the non-PBC cohort. Male PBC patients similarly had lower platelet counts and higher MELD, MELD-Na and MELD 3.0 scores, suggesting higher rates of portal hypertension and more severe disease.

Males with PBC had higher rates of complications compared with females apart from osteoporosis, which as expected, was significantly more likely to be present in females. Once again, these trends were not seen in the male non-PBC cirrhosis group, who had lower rates of most complications.

Concerningly, men with PBC were also found to be significantly younger on admission, with similar rates of most comorbidities, apart from higher rates of CKD, and lower rates of obesity. Before and after adjustment for age and comorbidities, male sex was independently associated with a much higher odds of inpatient mortality and liver transplantation, and longer durations of admission. They similarly had higher odds of clinically decompensated liver disease; however, this was not significant. These findings do not appear to be a feature of chronic liver disease in general, with non-PBC males instead having significantly lower odds of inpatient mortality and decompensation, but higher odds of liver transplantation – which, although non-significant, is in keeping with previous research demonstrating sex-based disparities in liver transplantation^16,17^..

Interaction term regression analysis further demonstrated that the impact of male sex on outcomes in PBC is significantly different to that in non-PBC, with considerably large effect sizes noted for the primary composite outcome of liver transplantation or death (OR 6.59) as well as the secondary outcomes.

In addition, 81.8% of admissions were female, and this approximate female-to-male ratio of 8:2 was higher than expected. Traditional studies report female-to-male ratios varying from 9:1 to as high as 22:1^3–6^, thereby suggesting men were overrepresented in inpatient admissions. This ratio was therefore compared against patients routinely seen by our hepatology department in the outpatient setting with PBC. A total of 1,012 such patients were identified, with 79.9% being female, thereby demonstrating a similar female-to-male ratio. These ratios may demonstrate the increasing recognition of PBC in recent times. (7, 8) It should be noted however, that a higher proportion of males with PBC under the care of our outpatient clinic would have an inpatient admission (29.1% vs. 18.9%), albeit not necessarily for issues relating to chronic liver disease.

The differences in outcomes seen in men and women with PBC have multiple possible explanations. One possibility is poorer engagement in the outpatient setting, with men attending less outpatient clinic reviews compared to women during the observation period in our institution (median encounters for men: 2 (IQR 1–4) vs. 3 (IQR 1–11) for women, *p*< 0.001). Another possibility is delays to diagnosis in men leading to poorer outcomes^14,18^. These delays could in turn be explained by men being less symptomatic with PBC prior to presentation^18–20^. Further, men may be less likely to receive the primary treatment for PBC, ursodeoxycholic acid (UDCA)^21^. It has also been suggested that men are less likely to respond to UDCA^19^, however other studies have not been able to replicate these findings^22^.

Another explanation may be a potential protective role of hormones such as oestrogen in the progression of PBC^23^. Oestrogen receptors are expressed in cholangiocytes, and their expression is more prominent in PBC compared to normal conditions^24^. Oestrogen deficiency may contribute to disease progression, as evidences by disease flares in post-partum periods and worsening or onset of symptoms during peri-menopausal or menopausal periods when oestrogen levels decline^25–28^. Blocking oestrogen in animal models using agents such as tamoxifen has been shown to decrease bile duct proliferation^24^, and the use of the oestrogen containing combined oral contraceptive pill has been linked to a lower risk of PBC^29^.

These findings may explain why men in our PBC cohort experienced worse outcomes than women. Prior research has also demonstrated worse outcomes for males with PBC with respect to mortality^10,12,30^but these findings have been inconsistent or have been limited in their assessment of disease severity^31–33^. Relative strengths of our study are the large number of male admissions, multiple measures of disease severity, sensitivity analysis demonstrating consistent findings and the inclusion of a matched cohort of non-PBC cirrhosis patients for comparison.

Limitations include the retrospective nature of this study, and the use of ICD-10-GM codes for identification of cases with PBC, other causes of cirrhosis and complications. Sex was recorded based on patient self-reporting, and unmeasured confounders such as alcohol consumption, age of diagnosis or duration of disease could not be accurately identified. Cause of death data was not available, as such all-cause mortality was used instead of liver-related mortality. Importantly, data on treatment prescription and adherence, such as for UDCA or other therapies, were not available. This limited the assessment of differential treatment access and response on the observed sex-based disparities in outcomes. Analysis was conducted on data from inpatients, thereby limiting generalizability to the broader PBC population.

This study contributes to the limited body of research into the impact of male sex on outcomes in PBC. It highlights the need for tailored diagnostic and management approaches, as well as close follow-up strategies for male patients who may experience worse outcomes. The disparity in outcomes may be secondary to diagnostic delays, lower rates of UDCA prescription and efficacy or a more severe pathological progression of PBC in men compared to women.

## Conclusion

Men with PBC experience worse outcomes than women across a spectrum of measures. These differences appear to be specific to PBC compared to other causes of cirrhosis, suggesting the pathological progression of PBC in males may be more severe than in females. Further research is required to decipher the underlying mechanisms and implications for clinical management.

## Supplementary Information

Below is the link to the electronic supplementary material.

Supplementary Material 1

## Data Availability

The data that support the findings of this study are available on request from the corresponding author. The data are not publicly available due to privacy or ethical restrictions.
