# Supplementary material for: The impact of male sex on outcomes in primary biliary cholangitis
Source: Sci Rep. 2026 Mar 31;16:10548. doi: 10.1038/s41598-026-44615-0 (PMC13039752; doi:10.1038/s41598-026-44615-0)
Supplement: Supplementary file 1 — Supplementary Material 1 [file 41598_2026_44615_MOESM1_ESM.docx]

**Supplementary Materials**

**Table S1: ICD-10-GM codes used**

| **Disease or complication** | ICD-10-GM |
| --- | --- |
| Primary Biliary Cholangitis | K74.3 |
| Cirrhosis | K70.3  K74.4 – K74.7 |
| **Aetiologies of Chronic Liver Disease** |  |
| Alcoholic Liver Disease | K70 |
| Metabolic Dysfunction Associated-Steatotic Liver Disease | K75.8 K76.0 |
| Hepatitis C | B18.2 |
| Hepatitis B | B18.0, B18.1 |
| Primary Biliary Cholangitis | K74.3 |
| **Complications** |  |
| Ascites | R18 |
| Hepatic Encephalopathy | K72.7 |
| Variceal Haemorrhage | I98.3 |
| Other GI bleeding | K92.0 – K92.2 |
| Hepatorenal Syndrome | K76.7 |
| Bacterial Peritonitis | K65 |
| Bacterial infection – other | A01 – A05 A2 – A4  B95, B96  G00, G01  J13 – J14  L00 – L03  M00  N30.0, N34, N39, N41.0, N45.0 |
| Hepatocellular Carcinoma | C22 |
| Portal Vein Thrombosis | I81 |
| Sarcopaenia | M62.50 |
| Osteoporosis | M80 |
| Malnutrition | E40-E46 |
| **Comorbidities** |  |
| Diabetes | E10 – E14 |
| Obesity | E65 – E68 |
| Ischaemic Heart Disease | I20 – I25 |
| Congestive Cardiac Failure | I50 |
| Chronic Obstructive Pulmonary Disease | J43 – J44 |
| Cerebrovascular Disease | I60 – I65 |
| Chronic Kidney Disease | N17 – N19 |

**Table S2: Baseline characteristics and outcomes in PBC and Matched Cohort**

| **.** | **PBC** | **Matched Cohort** | ***p*** |
| --- | --- | --- | --- |
|  |  |  |  |
| *n* | 940 | 940 |  |
| Age (median years (IQR)) | 61.03 (18.81) | 60.73 (18.61) | 0.76 |
| Sex=F n (%) | 769 (81.81) | 288 (30.64) | **<0.01** |
|  |  |  |  |
| **Comorbidities** |  |  |  |
| Diabetes | 21.81% | 21.17% | 0.78 |
| Obesity | 5.96% | 6.60% | 0.63 |
| Ischaemic Heart Disease | 10.85% | 10.43% | 0.82 |
| Congestive Heart Failure | 6.91% | 6.91% | 1.00 |
| Chronic Obstructive Pulmonary Disease | 3.19% | 3.30% | 1.00 |
| Cerebrovascular Disease | 2.77% | 2.87% | 1.00 |
| Chronic Kidney Disease | 9.79% | 9.47% | 0.88 |
| **Serum Blood Tests** |  |  |  |
| ALT (IU/L) | 32.00 (41.00) | 33.00 (31.00) | 0.71 |
| AST (IU/L) | 43.00 (46.00) | 52.00 (53.00) | **<0.01** |
| ALP (IU/L) | 139.00 (150.00) | 119.00 (101.50) | **<0.01** |
| GGT (IU/L) | 85.00 (146.00) | 120.00 (204.25) | **<0.01** |
| Bilirubin (mg/dl) | 0.86 (2.65) | 1.22 (2.32) | **<0.01** |
| International Normalised Ratio | 1.14 (0.28) | 1.30 (0.37) | **<0.01** |
| Albumin (g/L) | 32.20 (13.10) | 29.55 (11.4) | **<0.01** |
| Sodium (mmol/L) | 139.00 (5.00) | 138.00 (7) | **<0.01** |
| Platelets (x10^9^/L) | 183.00 (153.00) | 118.00 (109.00) | **<0.01** |
| **Complications of Liver Disease** |  |  |  |
| Ascites | 15.00% | 29.15% | **<0.01** |
| Hepatic Encephalopathy | 7.45% | 18.62% | **<0.01** |
| Variceal Haemorrhage | 2.55% | 5.64% | **<0.01** |
| Other Gastrointestinal bleeding | 1.60% | 4.04% | **<0.01** |
| Hepatorenal Syndrome | 2.87% | 8.40% | **<0.01** |
| Bacterial Peritonitis | 3.40% | 5.00% | 0.11 |
| Bacterial Infection - Other | 19.68% | 31.49% | **<0.01** |
| Hepatocellular Carcinoma | 4.15% | 18.30% | **<0.01** |
| Portal Vein Thrombosis | 1.49% | 3.94% | **<0.01** |
| Sarcopenia | 1.38% | 1.38% | 1.00 |
| Osteoporosis | 10.32% | 2.13% | **<0.01** |
| Malnutrition | 3.40% | 7.02% | **<0.01** |
| **Outcomes** |  |  |  |
| *Primary* |  |  |  |
| Liver Transplant or In-Hospital Mortality | 6.81% | 12.23% | **<0.01** |
| *Secondary* |  |  |  |
| In-Hospital Mortality | 3.09% | 10.11% | **<0.01** |
| Liver Transplant | 4.15% | 2.34% | **0.04** |
| Decompensated Cirrhosis | 19.30% | 40.43% | **<0.01** |
| Length of Admission (days) | 4.00 (7.00) | 6.00 (12.00) | **<0.01** |

**Abbreviations**: PBC = Primary Biliary Cholangitis; IQR = Interquartile Range.
p-values as determined by Mann-Whitney U, Fisher exact or Chi-Square tests

**Table S3: Assessing the balance post matching non-PBC cirrhosis controls to PBC**

| **Variable** | **Means PBC** | **Means Control** | **Std. Mean Difference** | **eCDF Mean** | **eCDF Max** |
| --- | --- | --- | --- | --- | --- |
| Age | 60.5862 | 60.3574 | 0.0169 | 0.0031 | 0.0138 |
| Diabetes | 0.2181 | 0.2117 | 0.0155 | 0.0064 | 0.0064 |
| Obesity | 0.0596 | 0.0660 | -0.0270 | 0.0064 | 0.0064 |
| Ischaemic Heart Disease | 0.1085 | 0.1043 | 0.0137 | 0.0043 | 0.0043 |
| Congestive Heart Failure | 0.0691 | 0.0691 | 0.0000 | 0.0000 | 0.0000 |
| Chronic Obstructive Pulmonary Disease | 0.0319 | 0.0330 | -0.0061 | 0.0011 | 0.0011 |
| Chronic Kidney Disease | 0.0979 | 0.0947 | 0.0107 | 0.0032 | 0.0032 |

**Abbreviations**: PBC = Primary Biliary Cholangitis, eCDF = empirical cumulative distribution function

**Table S4: Sensitivity Analysis: Impact of male sex on outcomes in primary biliary cholangitis - Generalised Estimating Equations with exchangeable correlation structure**

|  | **PBC** |  |  |  | **Matched Cohort** |  |  |  |
| --- | --- | --- | --- | --- | --- | --- | --- | --- |
| **Univariable** |  |  |  |  |  |  |  |  |
|  | OR | 95% CI LL | 95%CI UL | p | OR | 95% CI LL | 95%CI UL | p |
| Liver transplant or inpatient mortality | 4.28 | **2.53** | **7.23** | **<0.001** | 0.65 | **0.43** | **0.97** | **0.036** |
| Inpatient mortality | 2.45 | **1.12** | **5.37** | **0.025** | 0.54 | **0.35** | **0.84** | **0.006** |
| Liver transplant | 5.22 | **2.72** | **10.02** | **<0.001** | 2.03 | 0.68 | 6.05 | 0.204 |
| Decompensation | 1.42 | 0.95 | 2.13 | 0.084 | 0.74 | **0.56** | **0.98** | **0.036** |
|  | | | | | | | | |
| **Multivariable** |  |  |  |  |  |  |  |  |
| Liver transplant or inpatient mortality | 3.69 | **2.06** | **6.60** | **<0.001** | 0.63 | **0.42** | **0.95** | **0.029** |
| Inpatient mortality | 2.51 | **1.09** | **5.81** | **0.031** | 0.54 | **0.35** | **0.84** | **0.006** |
| Liver transplant | 3.36 | **1.56** | **7.25** | **0.002** | 2.52 | 0.82 | 7.80 | 0.108 |
| Decompensation | 1.19 | 0.78 | 1.82 | 0.415 | 0.74 | **0.55** | **0.98** | **0.034** |
|  | | | | | | | | |
| **Interaction analysis** | **Combined Cohort** | | | |  |  |  |  |
| Liver transplant or inpatient mortality | 6.60 | **3.40** | **12.78** | **0.000** |  |  |  |  |
| Inpatient mortality | 4.50 | **1.84** | **11.02** | **0.001** |  |  |  |  |
| Liver transplant | 2.59 | 0.73 | 9.25 | 0.142 |  |  |  |  |
| Decompensation | 1.84 | **1.13** | **3.00** | **0.014** |  |  |  |  |

**Abbreviations** PBC = Primary biliary cholangitis; OR = Odds ratio; CI = Confidence interval

**Table S5: Logistic and linear regression assessing the effect of male sex on outcomes in patients with PBC – sensitivity analysis on subset of patients with multiple indicators of PBC***

|  | **Univariable** | | | | | **Multivariable** | | | | |
| --- | --- | --- | --- | --- | --- | --- | --- | --- | --- | --- |
|  |  |  | **95% CI** | |  |  |  | **95% CI** | |  |
|  | **OR** | **β** | **Lower** | **Upper** | ***p*** | **OR** | **β** | **Lower** | **Upper** | ***p*** |
| ***Primary Outcome*** |  |  |  |  |  |  |  |  |  |  |
| Liver Transplant or Inpatient Mortality | 3.19 |  | **1.99** | **7.54** | **<0.001** | 3.68 |  | **1.64** | **8.16** | **0.001** |
| ***Secondary Outcomes*** |  |  |  |  |  |  |  |  |  |  |
| Inpatient Mortality | 1.43 |  | 0.32 | 4.76 | 0.59 | 2.05 |  | 0.43 | 7.59 | 0.309 |
| Liver Transplant | 4.87 |  | **2.26** | **10.43** | **<0.001** | 3.03 |  | **1.13** | **8.01** | **0.026** |
| Decompensation | 1.47 |  | 0.91 | 2.32 | 0.104 | 1.24 |  | 0.74 | 2.01 | 0.403 |
| Length of Admission* |  | 0.21 | **0.028** | **0.393** | **0.024** |  | 0.18 | -0.01 | 0.362 | 0.068 |

Covariables adjusted for in the multivariable model include age, diabetes, obesity, chronic obstructive pulmonary disease, chronic kidney disease. Length of admission log-transformed.
*Multiple ICD-10-GM PBC codes, AMA, GP100 or SP210 positivity where available, elevated ALP. Autoimmune Hepatitis cases excluded. Yielding 121 males and 573 females.

**Table S6: Assessing the balance post matching PBC females to PBC males**

| **Variable** | **Means PBC** | **Means control** | **Std. mean difference** | **eCDF mean** | **eCDF max** | **Std. pair distance** |
| --- | --- | --- | --- | --- | --- | --- |
| Age | 56.006 | 56.163 | -0.0102 | 0.0633 | 0.1266 | NA |
| Diabetes | 0.2405 | 0.2405 | 0 | 0 | 0 | NA |
| Obesity | 0.0253 | 0.0443 | -0.1037 | 0.0095 | 0.019 | NA |
| Ischaemic heart disease | 0.0949 | 0.0886 | 0.0219 | 0.0032 | 0.0063 | NA |
| Congestive heart failure | 0.0506 | 0.0696 | 0.0799 | 0.0095 | 0.019 | NA |
| Chronic obstructive pulmonary disease | 0.0063 | 0.019 | -0.1134 | 0.0063 | 0.0127 | NA |
| Chronic kidney disease | 0.1329 | 0.1646 | -0.089 | 0.0158 | 0.0316 | NA |

**Abbreviations**: PBC = Primary Biliary Cholangitis, eCDF = empirical cumulative distribution function, NA = not applicable

**Table S7: Logistic and linear regression assessing the impact of male sex on outcomes in the matched PBC cohort (PBC males and females matched on a 1:1 ratio)**

|  |  |  | **95% CI** | |  |
| --- | --- | --- | --- | --- | --- |
|  | **OR** | **β** | **Lower** | **Upper** | ***p*** |
| ***Primary Outcome*** |  |  |  |  |  |
| Liver Transplant or Inpatient Mortality | 3.05 |  | **1.47** | **6.84** | **0.004** |
| ***Secondary Outcomes*** |  |  |  |  |  |
| Inpatient Mortality | 1.53 |  | 0.54 | 4.66 | 0.430 |
| Liver Transplant | 3.46 |  | **1.42** | **9.73** | **0.010** |
| Decompensated Cirrhosis | 1.25 |  | 0.73 | 2.13 | 0.417 |
| Length of Admission (days) |  | 0.13 | -0.10 | 0.35 | 0.26 |

In a cohort of 171 male cases and 171 matched female cases. Matching performed based on age and comorbidities. Results displayed are for univariable analysis without further adjustment of covariables post matching. **Abbreviations:** PBC = Primary biliary cholangitis; CI = Confidence interval; OR = Odds ratio.
